# Potential Use of Exhaled Breath Condensate for Diagnosis of SARS-CoV-2 Infections: A Systematic Review and Meta-Analysis

**DOI:** 10.3390/diagnostics12092245

**Published:** 2022-09-17

**Authors:** Matteo Riccò, Alessandro Zaniboni, Elia Satta, Silvia Ranzieri, Federico Marchesi

**Affiliations:** 1 Servizio di Prevenzione e Sicurezza Negli Ambienti di Lavoro (SPSAL), AUSL-IRCCS di Reggio Emilia, Via Amendola n.2, I-42122 Reggio Emilia, Italy; 2 Department of Medicine and Surgery, University of Parma, Via Gramsci, 14, I-43126 Parma, Italy

**Keywords:** exhaled breath condensate, COVID-19, SARS-CoV-2, airborne pathogens, respiratory airways

## Abstract

Background. Reverse-transcriptase polymerase chain reaction (RT-qPCR) assays performed on respiratory samples collected through nasal swabs still represent the gold standard for COVID-19 diagnosis. Alternative methods to this invasive and time-consuming options are still being inquired, including the collection of airways lining fluids through exhaled breath condensate (EBC). Materials and Methods. We performed a systematic review and meta-analysis in order to explore the reliability of EBC as a way to collect respiratory specimens for RT-qPCR for diagnosis of COVID-19. Results. A total of 4 studies (205 specimens), were ultimately collected, with a pooled sensitivity of 69.5% (95%CI 26.8–93.4), and a pooled specificity of 98.3% (95%CI 87.8–99.8), associated with high heterogeneity and scarce diagnostic agreement with the gold standard represented by nasal swabs (Cohen’s kappa = 0.585). Discussion. Even though non-invasive options for diagnosis of COVID-19 are still necessary, EBC-based RT-qPCR showed scarce diagnostic performances, ultimately impairing its implementation in real-world settings. However, as few studies have been carried out to date, and the studies included in the present review are characterized by low numbers and low sample power, further research are requested to fully characterize the actual reliability of EBC-based RT-qPCR in the diagnosis of COVID-19.

## 1. Introduction

Exhaled breath condensate (EBC) is a fluid or frozen material matrix obtained by cooling exhaled air on the cold surfaces of a condenser [1,2]: resulting fluids are mostly water vapor, but a small fraction contains respiratory droplets from the airway lining fluids [3], including volatile, slightly volatile (i.e., carbon dioxide, nitric oxide, hydrocarbons), and even non-volatile substances (i.e., proteins, lipids, electrolytes, acidic and alkaline small molecules, metals) [4,5,6,7].

Previous studies have suggested that EBC may represent a non-invasive option for collecting microbial specimens from the airways, as an alternative to the collection of biological samples from nasal swabs, bronchoalveolar lavage (BAL), nasopharyngeal aspirates, and sputum [3,8]. More precisely, some studies have detected viral RNA in EBC collected from subjects affected by influenza virus, rhinoviruses, and most notably human coronaviruses [9,10,11]. Moreover, some studies have suggested that EBC may be collected even from patients undergoing noninvasive ventilatory support [12,13]. While earlier studies have stressed the feasibility of microbiological analyses, and particularly the processing of collected specimens by means of the real time quantitative polymerase chain reaction (RT-qPCR) [3,8,9,10,11], its reliability for diagnosis of respiratory infections in real-world settings still remains doubtful because of an unsatisfying detection rate [3].

More recently, the SARS-CoV-2 pandemic has substantially renewed the interest towards the potential referral to EBC as diagnostic matrix for viral pathogens, and particularly for SARS-CoV-2 [4,13,14,15,16,17,18]. For instance, a study from late 2020 has showed evidence that SARS-CoV-2 can be detected in EBC by means of RT-qPCR [4,19], prompting an increasing interest towards the use of alternative methods such as lateral flow assays or ELISA technologies [5,6]. EBC collection is relatively simple (i.e., the subject’s breath only need to be directed over a cold surface) [1], far more comfortable than nasal swabs, easily scalable by either interventions on the procedures (i.e., longer collection time and/or higher minute ventilation substantially increase the amount of collected specimen) or on the characteristics of the samples (e.g., by increasing the size of the cold surfaces) [1,2], and may be performed either with homemade or commercial equipment [20]. Moreover, as EBC retrieves specimens from the whole surface of the airways [3,4], it has the potential to improve the detection rate in respect to more conventional approaches more extensively based on the proximal respiratory tract [19]. Specimens from the proximal respiratory tracts (including saliva, nasal, anterior nares swabs, oropharyngeal and nasopharungeal swabs) are relatively easy to acquire but could miss areas with high viral loads during the swabbing or may be affected by viral load fluctuations, leading to false-negative tests results [19,21]. For example, in a study on 2413 patients with an early stage COVID-19 infection, 18.6% of nasopharyngeal specimens returned a negative RT-qPCR result, that in presence of suggesting clinical features resulted in a positive result at a follow-up retest performed within 24 h [19,22].

While previous studies have reported highly inconsistent results [4,13,14,15,16,17,18,21,23,24], there is a substantial lack of evidence on the referral to EBC collection on diagnosis of COVID-19. An updated synthesis of the literature may be therefore useful to healthcare professionals involved in the research of new, non-invasive options for the diagnosis of SARS-CoV-2 infections. As a consequence, the present systematic review and meta-analysis was undertaken with the aim of exploring the reliability of EBC collection for PCR detection of SARS-CoV-2 in the management of the COVID-19 pandemic.

## 2. Materials and Methods

### 2.1. Search Strategy

The present systematic review and meta-analysis of the literature has been conducted according to the “Preferred Reporting Items for Systematic Reviews and Meta-Analysis” (PRISMA) guidelines [25] (PROSPERO registration number: CRD42022352431) [25]. Search questions were preventively determined according to the “PICO” (Patient/Population/Problem; Intervention; Control/Comparator; Outcome) strategy, modified for diagnostic tests (Table 1) [26].

Three different information sources (i.e., scholarly databases PubMed/MEDLINE and EMBASE, and the pre-print servers medrxiv.org) have been inquired for relevant studies from inception up to 28 February 2022, without applying any backward chronological restrictions. Reference lists of relevant articles were also assessed in order to retrieve further studies not identified from the initial research.

The search strategy was defined by a combination of the following keywords (free text and Medical Subject Heading [MeSH] terms, where appropriate): (“exhaled breath” OR “breath tests” OR “exhaled breath condensate” OR “breathomics”) AND (“COVID-19” OR “SARS-CoV-2” OR “Coronavirus”) AND (“diagnostic” OR “diagnostic test” OR “screening”).

### 2.2. Eligibility Criteria

Original research publications available online or through inter-library loan were considered eligible for review if:

1. Written in Italian, English, German, French or Spanish, i.e., the languages spoken by the investigators;
2. Reporting on original results, with the exclusion of reports, case series, meeting reports and conference abstracts;
3. Reporting data on human samples;
4. Dealing with COVID-19 cases diagnosed by means of conventional RT-qPCR tests on nasopharyngeal swabs [27];
5. Reporting the raw number of true positive/true negative, and false positive/false negative results;
6. Assessing the diagnostic accuracy of EBC in the diagnosis of SARS-CoV-2 infection.

Studies were also excluded if the analyses did not include a RT-qPCR for SARS-CoV-2 on EBC, or the RT-qPCR was performed on biological matrices other than EBC.

### 2.3. Study Selection and Data Extration

Records were initially screened by title and abstract by two independent authors (E.S. and A.Z.) about the eligibility criteria, and articles that met all of the inclusion criteria were then retained for the full-text review, which was independently performed by both investigators. Disagreements were resolved by consensus between the two reviewers; when it was not possible to reach consensus, input from a third investigator (M.R.) was searched and we obtained all articles that appeared to be pertinent to the search strategy were handled through a references management software (Mendeley Desktop Version 1.19.5, Mendeley Ltd., London, UK, 2019). Data extracted included:

1. Settings of the study;
2. Characteristics of reference cases;
3. Characteristics of the EBC collecting device;
4. Total number of true positive, true negative, false positive, false negative cases.

### 2.4. Quality Assessment

Qualitative assessment of the studies was performed by two review authors (E.S. and A.Z.) independently assessing methodological quality by means of the Quality Assessment of Diagnostic Accuracy Studies (QUADAS-2) [28]. This tool comprises 4 domains: patient selection, index test, reference standard, and flow and timing. Each domain is assessed in terms of risk of bias, with the first 3 domains that are also assessed in terms of concerns regarding their applicability. The quality assessment results were then classified into low concern, some concerns, and high concerns in terms of risk bias, and plotted into specific charts.

### 2.5. Quantitative Analysis

Included studies were initially summarized through a descriptive analysis that, by assuming RT-qPCR on nasal swabs as gold standard reported corresponding rates for True Positive, False Negative, False Positive, and True Negative cases. For each study, sensitivity (Se; i.e., the proportion of positive cases among people with a given disease), specificity (Sp; i.e., proportion of negative cases among people without that disease), positive and negative likelihood ratio (PLR and NLR), diagnostic odds ratio (DOR), accuracy and Cohen’s “kappa” were then calculated as follows.

By definition, PLR is the probability that a subjected affected by a certain disorder do result positive from a certain test divided the probability that the test would be result positive at that testing in a patient without the disease (i.e., PLR = Se/(1 − Sp). Conversely, NLR has been defined as the probability of obtaining a negative result while actually having a certain disease divided by the probability of testing negative while not having that certain disease (NLR = (1 − sensitivity)/Sp).

DOR is a single measure of diagnostic test performances. It expresses how much greater the odds are of having the assessed condition in subjects with a positive test result compared to people with a negative test result. In this study, it was calculated by dividing PLR by NLR.

The accuracy of any test may be acknowledged as its ability to correctly differentiate the patient and healthy cases. It can be estimated by comparing the results from a certain diagnostic test with those from a reference one (i.e., gold standard; in this case, presence or absence of SARS-CoV-2 nucleic acid at RT-qPCR on nasal swabs).

Cohen’s kappa coefficient quantitatively assesses inter-rater reliability of qualitative (i.e., categorical) items as the agreement between two tests or raters. Cohen’s kappa values are usually categorized as reporting “weak” to “none” agreement for kappa < 0.600; “moderate” agreement for values ranging 0.600 to 0.799; “strong” agreement for values ranging between 0.800 and 0.900 “strong” agreement. Values of kappa > 0.900 are usually acknowledged for “almost perfect” agreement.

Pooled estimates for Se, Sp, PLR, NLR, accuracy, Cohen’s kappa and DOR were calculated through a random-effects model that combined each study’s results.

In order to cope with the threshold effect, i.e., the potential heterogeneity in the estimates for Se and Sp resulting from different cut-offs used in different diagnostic kits, summary receiver operating characteristic (sROC) curves were specifically calculated by plotting accuracy estimates from included studies in two distinctive models (i.e., a bivariate model vs. a fixed, unweighted, model). The overlapping of sROC curves will suggest absence of threshold effect, that could be conversely acknowledged for substantial differences at visual inspection [29].

The inconsistency between included studies was estimated by calculation of I^2^ statistics. I^2^ represents an estimate of the percentage of total variation across studies that is due to heterogeneity rather than to chance. In this study, I2 was calculated through a fixed-effects model because of the reduced number of samples eventually included, and following ranges were acknowledged: low heterogeneity for I^2^ values ranging from 0% to 25%; moderate heterogeneity for I^2^ values ranging from 26% to 50%; substantial heterogeneity for estimates above 50%.

Small study bias was then ascertained through calculation of radial plots and their subsequent visual inspection. Eventually, publication bias was ascertained by means of contour-enhanced funnel plots where sample size was plotted against effect size. Funnel plots were visually inspected in order to ascertain their asymmetry, suggesting potential publication bias. Moreover, Eggers’ test (i.e., a linear regression of effect estimates divided by its standard error against reciprocal of the standard error of the estimate) was performed for quantitative publication bias analysis (at a 5% of significance level). In fact, Egger’s test may yield false positive results if fewer than 10 studies were included.

All calculations were performed in R (version 4.1.1; R Core Team, 2017. R: A language and environment for statistical computing. R Foundation for Statistical Computing, Vienna, Austria. URL https://www.R-project.org/, accessed on 1 July 2022) [30], and RStudio (version 2021.09.0 build 351, RStudio PBC, 250 Northern Ave, Boston, MA, USA) software by means of *robvis* (version 0.3.0), *meta* (version 5.2-0), *mada* (version 0.5-10), and *nsROC* (version 1.1) packages. All packages are open-source add-ons for conducting meta-analyses and systematic reviews.

## 3. Results

**Figure 1:**
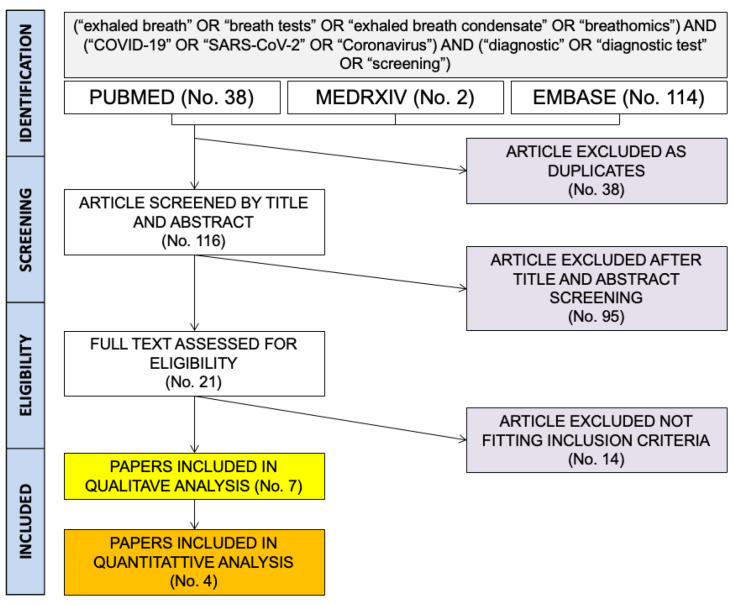
The process of studies retrieval and inclusion adopted in the present systematic review and meta-analysis. A total of 7 studies were included in qualitative analysis, and 4 of them were summarized in quantitative analysis.

A total of 154 entries (i.e., 38 from PubMed; 2 from MedRxiv; 114 from EMBASE) were initially retrieved (Figure 1).

After the removal of duplicates (No. 38), 116 articles were screened by title and abstract. Of them, 95 were removed after the title and abstract screening. Twenty-one articles were then assessed and reviewed by full-text. Eventually, 7 papers [4,13,14,16,17,18,31] were included in qualitative analysis (Table 2). The study from Viklund et al. [18] included a total of 2 estimates, but one of them was excluded from both qualitative and quantitative analysis as the confirmatory test was a rapid antigen test rather than a RT-qPCR based one.

Overall, the systematic review included 361 samples (219 of them SARS-CoV-2 positive as confirmed by RT-qPCR; 60.7%) from 4 estimates, each of them ranging from 17 [13] to 105 [14]. Of the retrieved samples, the retrieved estimates were based on a total of 6 different tests, 5 of them commercially available, and 1 study based on a “homemade” tester [31].

Of the aforementioned studies, three [13,16,31] were performed only on positive cases. As this would have impaired the proper calculation of Sp estimates, such reports were eventually excluded from metanalysis, while the remaining 4 studies with 205 samples were eventually included in quantitative analyses [4,14,17,18] (Table 3).

**Figure 2:**
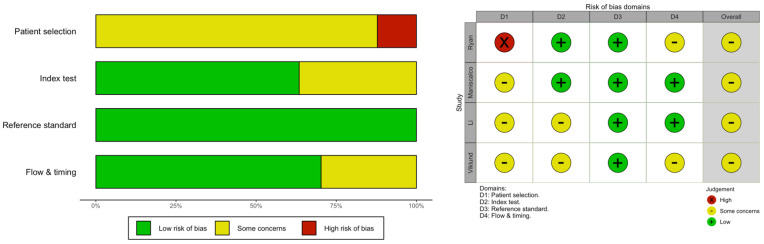
A QUADAS-2 assessment on the risk of bias.

Methodological quality assessment is summarized in Figure 2. All studies had an unclear [14,17,18] or even a high [4] risk of bias because of the inpatient selection, as it was quite unclear whether participants were actually comparable in terms of stage of the infection. Regarding the index test, two studies [17,18] reported an unclear risk of bias because of the characteristics of the device that in one case was home-made and specifically designed for the present study. Regarding the reference standard, the risk of bias was substantially low in all studies, as by design the present meta-analysis did include only studies having RT-qPCR as a confirmatory test. Eventually, when dealing with flow and timing, two studies were potentially affected by some issues as the timing between index tests and reference standard was unclearly reported [4,18].

A summary of the diagnostic performances of the RT-qPCR tests performed on EBC is reported in Table 4.

**Figure 3:**
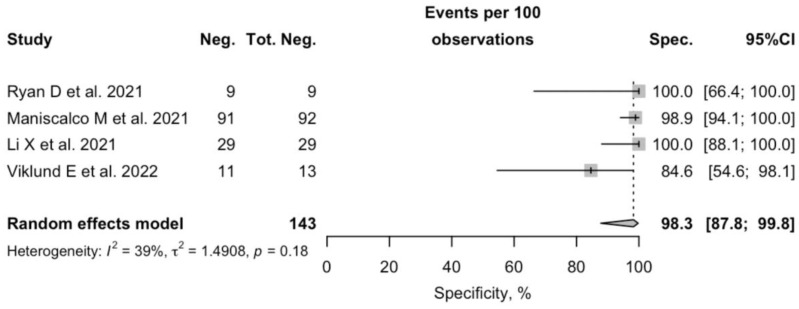
A forest plot representing the estimated specificity of SARS-CoV-2 Real-Time Quantitative Polymerase Chain Reaction (RT-qPCR) performed on specimens of exhaled breath condensate. Pooled specificity was 98.3% (95%CI 87.8–99.8), with moderate heterogeneity (I^2^ 39.0, *p* = 0.178) [4,14,17,18].

More precisely, Sp among reported studies ranged between 84.6% to 100% (pooled Sp 98.3%; 95%CI 87.0–99.8). As shown in Figure 3, the heterogeneity was moderate (I^2^ 39.0%, *p* = 0.178).

**Figure 4:**
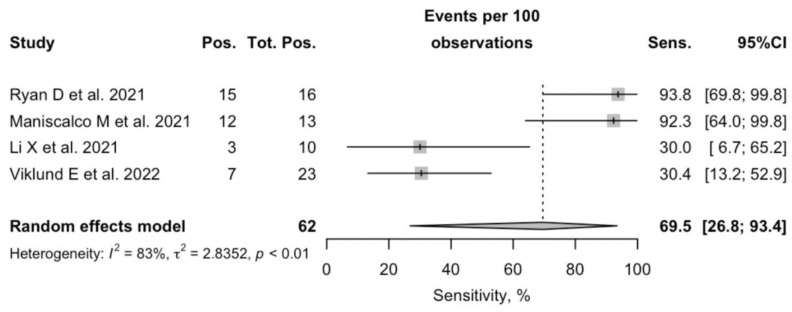
A forest plot representing the estimated sensitivity of SARS-CoV-2 Real-Time Quantitative Polymerase Chain Reaction (RT-qPCR) performed on specimens of exhaled breath condensate. Pooled sensitivity was 69.5% (95%CI 26.8–93.4), and heterogeneity was substantial (I^2^ 82.7%, *p* = 0.006) [4,14,17,18].

On the contrary (see Figure 4), Se ranged from 30.0% to 93.8 (pooled Se 69.5%; 95%CI 26.8–93.5), that was otherwise affected by substantial heterogeneity (I^2^ 82.7%, *p* = 0.006).

Effectiveness of the RT-qPCR on EBC specimens compared to conventional nasal swabs was then assessed by calculating pooled PLR, NLR, DOR and Cohen’s Kappa values. Pooled PLR was estimated to 23.608 (95%CI 20.752 to 26.464), with a corresponding NLR of 0.433 (95%CI 0.384 to 0.482). In other words, positive tests were associated with relatively strong evidence of actual infection, while negative ones had the moderate chance of being actually affected by SARS-CoV-2 infection. Moreover, estimates for PLR and NLR were plotted in Fagan’s nomogram in order to ascertain the corresponding post-test performances of the test (i.e., the probability of the patient actually having a disease after obtaining the test results) by plotting corresponding pretest probability (i.e., the probability that an individual has the condition ascertained by a certain test before he/she was tested) [23].

**Figure 5:**
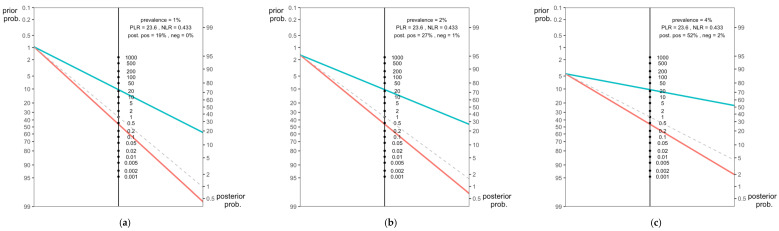
Fagan’s nomograms for SARS-CoV-2 Real-Time Quantitative Polymerase Chain Reaction (RT-qPCR) were performed on specimens of exhaled breath condensate. In the model, actual prevalence estimates for SARS-CoV-2 in Italy were included from 3 different time periods: (**a**) 31 December 2020 (1%), (**b**) 31 December 2021 (2%), and (**c**) 31 January 2022 (4%). Assuming that RT-qPCR on exhaled breath condensate has an estimated diagnostic sensitivity (Se) of 69.5% and specificity (Sp) of 98.3%, with corresponding estimates for PLR and NLR respectively of 23.608 and 0.433, the post-test probability that a positive case is truly infected by SARS-CoV-2 would be approximately 19% (blue line) for 1% prevalence, 27% for 2%, and 52% for 4% prevalence. Alternatively, if the patient tests negative, the post-test probability that he/she is truly infected by SARS-CoV-2 would be approximately 0% (red line) for 1% prevalence, 1% for 2% prevalence, 2% for 4% prevalence.

Estimates were calculated according to reported prevalence rates for SARS-CoV-2 in Italy on 31 December 2020 (i.e., 1% of total population), 31 December 2021 (i.e., 2% of total population), and 31 January 2022 (i.e., 4% of total population), and post-test actual performances ranged between 19% (31 December 2020) to 27% (12 December 2021), and eventually 52% (31 January 2022) (Figure 5).

**Figure 6:**
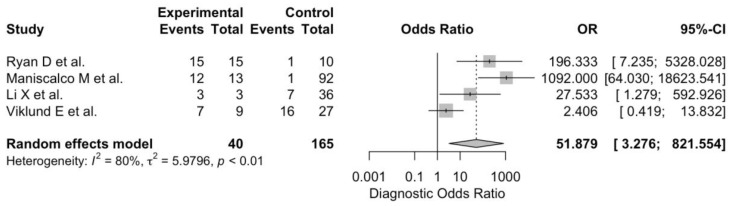
A forest plot representing the pooled estimate of diagnostic odds ratio (DOR) for SARS-CoV-2 Real-Time Quantitative Polymerase Chain Reaction (RT-qPCR) performed on specimens of exhaled breath condensate. Pooled DOR was 51.879 (95%CI 3.276 to 821.554), and heterogeneity was substantial (I^2^ 80.2%, *p* = 0.002) [4,14,17,18].

Correspondent pooled DOR from the retrieved studies was 51.879 (95%CI 3.276 to 821.554). In other words, individuals with the disease had substantially greater odds of a positive test compared with those without the disease but still positive at RT-qPCR. However, as shown in Figure 6, heterogeneity was substantial (I^2^ = 80.2%, *p* = 0.002). Eventually, the pooled accuracy was 81.5% (95%CI 78.7% to 84.3%), with a pooled estimate for Cohen’s kappa equals to 0.585 (95%CI 0.535 to 0.635). In other words, despite a substantial heterogeneity (I^2^ 99.9% in both cases), the agreement between EBC-based and nasal-swab based RT-qPCR tests may be assumed to be moderate.

**Figure 7:**
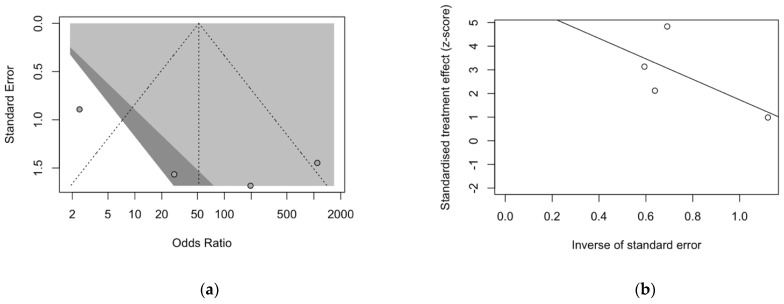
Funnel plots for DOR studies included in the metanalysis (**a**). Visual inspection of funnel plot suggested a significant asymmetry for all analyses, with subsequent reporting bias. Visual inspection of radial plot suggests a seemingly random distribution of included studies on both sides of the regression line. As otherwise suggested by radial plot (**b**) regression analysis dismissed a significant reporting bias (*t* = 2.11, df = 2, *p*-value = 0.1689).

Keeping in mind the very small number of included studies, visual inspection of contour-enhanced funnel plots for DOR (Figure 7) was consistent with a significant asymmetry for all analyses, with subsequent reporting bias, that was otherwise dismissed at regression analysis (*t* = 2.11, df = 2, *p*-value = 0.1689). In fact, the corresponding radial plot was also characterized by a seemingly random distribution of the studies across the regression line, reasonably ruling out the possible small-study effect on the overall estimates.

**Figure 8:**
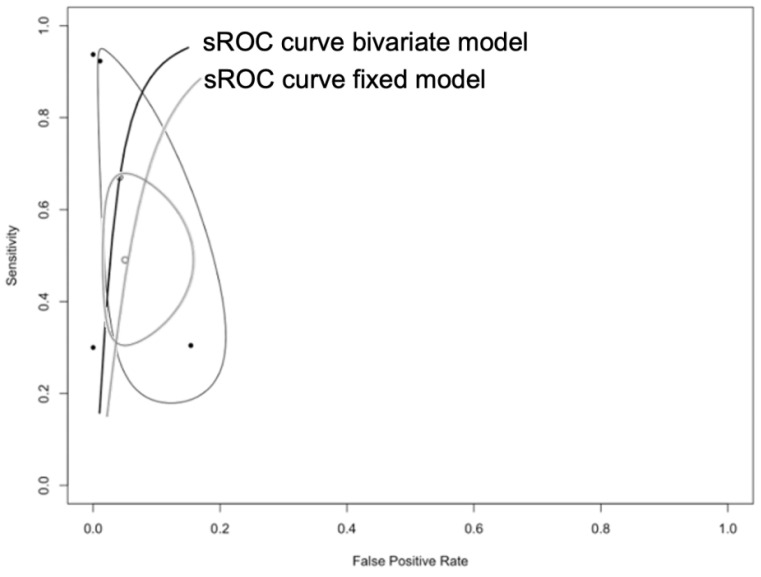
Summary Receiver Operated Characteristics (sROC) curves for PCR testing performed on exhaled breath condensate compared to conventional nasal swabs. The noticeable differences between estimates from a random-effect (bivariate) model and a fixed-effect model are consistent with the hypothesis of a threshold effect in diagnostic performances of assessed tests.

Eventually, sROC curves were calculated, with a noticeable difference between the bivariate model (Area Under Curve [AUC] = 0.953) and the fixed model AUC = 0.774). In other words, a threshold effect was actually affecting reported results (Figure 8).

## 4. Discussion

In this systematic review and meta-analysis, we reported on the diagnostic performances of RT-qPCR performed on specimens collected by means of EBC compared to conventional samples from nasal swabs. In fact, only a reduced number of studies (No. 4), with a limited number of specimens (No. 205) were ultimately evaluated [4,14,17,18]. Despite some earlier and promising remarks [4,17,21,23,31], pooled sensitivity (69.5%, 95%CI 26.8 to 93.4), as well as the diagnostic agreement between RT-qPCR assays rather performed on EBC than on the gold standard represented by nasal swabs (0.585, 95%CI 0.535 to 0.635) were clearly far from optimal [32,33,34]. In fact, such estimates were in line with earlier reports suggesting the alternative and innovative diagnostic procedures (e.g., Highly Sensitive High Performance Liquid Chromatography Laser Induced Fluorescence, photoacoustic spectroscopy and e-Nose) may represent a more rational choice to be coupled with EBC [5,6]. Nevertheless, diagnostic performances were substantially in line with those guaranteed by rapid antigenic testing [35,36,37], whose costs in terms of infrastructural, human and monetary resources are hardly comparable with the requests of EBC collection [2,20,38]. Moreover, application of Fagan’s nomogram [39] in order to cope with requirements of real-world settings hinted towards a further loss of diagnostic performances facing a lower circulation of the pathogen in the reference population. In other words, EBC-based studies could fail in detecting false negative cases when this possibility would be particularly appreciated in order to guarantee the early detection of incident cases, ultimately avoiding new outbreaks in a vulnerable population.

Our results may appear unsatisfactory, but some explanations may be found in both the characteristics of retrieved studies and the general specificities of EBC. For one, as suggested by calculation of sROC curves, and consistently with our current understanding of SARS-CoV-2 diagnostic options, EBC appeared particularly vulnerable to a threshold effect [27]. In other words, similarly to rapid antigen testing assays, whose reliability may be disputed for replicatory activity above the RT-qPCR equivalent of 25 to 30 cycle threshold [34,35,36,37], our data hints towards a scarce trustworthiness of EBC in cases of reduced availability of viral RNA. In turn, the potential shortcoming might be explained through specificities of collecting devices, strategies, and sampled patients.

More precisely, studies characterized by higher sensitivity estimates seemingly rely on more conventional testers that have been specifically designed for EBC collection [4,14], while lesser performing tests were either based on “homemade” devices [17], or on partially repurposed ones [18]. Hence, we cannot rule out that the scarce performances of EBC-based RT-qPCR might be a consequence of the scarce amount of collected viral RNA through scarcely efficient devices, and this potential issue could be solved by further comparisons between available testers, similarly to real-world studies on point-of-care tests [27,37]. On the contrary, the potential contamination or, conversely, the dilution of the collected EBC by saliva or other proximal respiratory fluids may be substantially excluded in the design of collecting devices [4,14,17,18].

Even regarding the collection of the samples, the studies were quite heterogenous. For example, in the study by Ryan et al. [4], participants were requested to breath for a fixed time (2 min) through the mouthpiece, while in the studies by Li et al. [17], Viklund et al. [18], and Maniscalco et al. [14] the target was represented by the amount of collected EBC. Eventually, while in three out of the included 4 studies participants were requested to only perform regular breaths [4,14,17], in the study by Viklund et al. [18] participants also performed normal breathing, airway opening maneuver, and cough.

Similarly, the clinical characteristics of the sampled patients were limitedly comparable. For instance, Viklund et al. [18] included a total of 36 samples from healthcare workers during medical surveillance, while the study of Li et al. [17] reported on a large share of individuals that were either in later stages of their disease or were scarcely symptomatic. On the contrary, the larger study of Maniscalco et al. [14] (No. = 105, i.e., more than half of the whole pooled population included in the quantitative analysis) relied on a more diverse set of patients, ranging from asymptomatic ones, with low clinical probability (47.6% of sampled population), to clinically suspected COVID-19 ones (20.0%). Similarly, Ryan et al. [4] included in their estimates cases that were clinically suspected for SARS-CoV-2 infection (31 out of 40 sampled patients, i.e., 77.5%). As a consequence, we cannot rule out the potential oversampling of individuals with active viral replication in the studies where better estimates were identified [4,14].

Nonetheless, there is some evidence that the timing of viral replication of SARS-CoV-2 is not homogenous across the affected airways [40,41], with a resulting diachronous detection of this pathogen across various types of clinical specimens. Even though earlier studies on BAL hinted towards a better proficiency of this specimens (93%) compared to nasal swabs (63%) and pharyngeal swabs (32%), it should be stressed that the former specimens were mostly collected from more severe cases, characterized by a significant involvement of deeper airways [41]. In individuals where a preventive interaction with the pathogen, either guaranteed by natural infection or vaccination, eventually leads to less invasive infections, airways and mostly bronchiole may be only limitedly affected by viral invasion [42,43,44]. Lining fluids, in turn, will be only limitedly laden with viral nucleic acids, leading to the poor performances of RT-qPCR because of scarcity or even the absence of SARS-CoV-2 RNA. It is important to stress that this potential issue is nothing new to the EBC-based diagnostic strategies, as previously reported in various studies on respiratory pathogens, representing a well-known and diffusely acknowledged limit to this diagnostic option [1,3,20].

*Limits*. Albeit interesting, our study is affected by substantial shortcomings that must be clearly acknowledged. To begin with, we must acknowledge the implicit limits of all meta-analyses, as quantitative estimates are forcibly dependent on the quality and heterogeneity of the source studies [45,46]. As previously stressed, not only we were able to retrieve a small number of studies, but also their content was strikingly heterogeneous in terms of overall quality and the targeted populations. Second, because of the lack of detailed reporting from source material, our meta-analysis was unable to take in account the delay between the reported onset of the symptoms (if any) and the actual testing. As a consequence, it is possible that even collecting devices with less satisfying performances may have been further impaired from the status of index patients, underestimating their actual sensitivity in optimal settings. Therefore, our results should be cautiously considered, by stressing that our study was not designed in order to represent a comparative assessment of diagnostic kits and/or devices. Third, all the studies that were included in the quantitative analysis were generally characterized by low numbers and low sample power; moreover, one of them included nearly half of the pooled samples, limiting the overall reliability of reported estimates [14]. Eventually, all the reported studies were performed before the emergence of Omicron variant of concern of SARS-CoV-2 and the high dependency of EBC-based RT-qPCR on the viral nucleic acid content in the collected fluids may represent an even greater limit when dealing with these insidious pathogens. Compared to the ancestral Wuhan strain, Omicron and its subvariants have been allegedly associated with milder clinical features, that in turn appear to be associated with a different pattern of viral invasion [47,48,49]. In fact, Omicron strains seems more efficient in replicating within the mucosa of upper airways [50,51]: coupled with the higher efficiency in promoting breakthrough infections, this feature explains its considerably higher transmission [48,49,52,53], being also linked to some different features [48,53,54]. Being less invasive to the deeper airways, SARS-CoV-2 could be therefore less represented in lining fluids, leading to failed diagnosis when the study is performed through EBC compared to more conventional options such as nasal swabs, with obvious consequences.

## 5. Conclusions

In conclusion, despite its potential advantages, the use of EBC-based RT-qPCR for the diagnosis of SARS-CoV-2 infection cannot be, to date, recommended for clinical purposes and cannot substitute other more reliable conventional diagnostic tests, such as assays based on the nasal swabs. Similarly, future studies could more extensively address the reliability of biomarkers as an alternative to nucleic acids, allowing for the use of innovative diagnostic procedures based on the recognition of volatile organic compounds. Still, the present review was affected by significant shortcomings, including a reduced number of studies with small sample sizes of heterogeneous quality and design. Therefore, improved, high-quality research in the field is warranted for definitively ruling out a potential role of EBC in the management of SARS-CoV-2 diagnosis.

## Figures and Tables

**Table 1:** A PICO worksheet modified for studies on diagnostic tests [26] (Note: EBC = exhaled breath condensate; RT-qPCR = polymerase chain reaction test).

Item	Definition
Population of interest	Adults with suspected diagnosis of SARS-CoV-2 infection
Investigated test result	Results on RT-qPCR testing for SARS-CoV-2 infection performed on specimens collected as EBC
Comparator test result	Results on RT-qPCR testing for SARS-CoV-2 infection performed on specimens collected by means of nasal swabs
Outcome	SARS-CoV-2 positive status (or not)

**Table 2:** A summary of the studies included in the present meta-analysis (Note: EBC = exhaled breath condensate) [4,13,14,16,17,18,31].

Reference	Location (Settings)	Design	Characteristics of the Samples	Tester	Sample Collection
Ryan et al. 2021 [4]	Ireland (Hospital)	Prospective Observational Single center	Convenience sample; 40 patients including: 16 NPS positive, 15 NPS negative but clinical diagnosis of COVID, 9 negative cases with another respiratory disorder	RTube Condenser (Commercial)	Participants breathed for 2 min through the mouthpiece.
Maniscalco et al. 2021 [14]	Italy (Hospital)	Prospective Cross-Sectional Multicenter (2 independent hospitals)	Convenience Sample; 4 groups of subjects (i.e., 1 clinically suspected COVID19; 2 convalescent COVID19; 3 asymptomatic individuals at risk for COVID19; 4 asymptomatic individuals not at risk for COVID19)	Inflammacheck (Commercial)	Participants breathed into a disposable breath collection unit until enough condensate is formed (20–30 µL; usually 45 to 90 s)
Li et al. 2021 [17]	China (Laboratory + Hospital)	Prospective Proof of Concept Cross-Sectional Multicenter (2 independent hospitals)	Convenience 27 COVID-19 cases 12 healthy volunteers	In house	Participants breathed into a exhalation tube connected to a sampling head, that in turn is connected to a collection bottle, until enough condensate is collected (1.5 mL; usually 3–5 min)
Viklund et al. 2022 [18]	Sweden (Hospital)	Prospective Observational Single center	Convenience Samples from Healthcare workers either tested positive (No. 25) or negative (No. 11) at medical surveillance.	PExA AB (Commercial)	Participants inhaled air through a high-efficiancy particle arresting (HEPA) filter to remove ambient particles in order to remove external particles, then breathed through a mouthpiece. Three procedures were performed (normal breathing, airway opening maneuver; cough) with collection of 1.5 mL (time not specified).
Sawano et al. 2021 [16]	Japan (Hospital)	Prospective Observational Single center	Convenience sample, 50 patients with a previous diagnosis of COVID-19	RTube/R-tube Vent Condenser (Commercial)	Participants breathed freely through the mouthpiece to collect 0.5–1.0 mL of EBC (usually, 5 to 7 min)
Loconsole et al. 2022 [13]	Italy (Hospital)	Prospective Observational Single center	Convenience Sample from ICU with ARDS; 17 consecutive patients	Turbo DECCS System (Commercial)	Within 24 h from the collection of conventional specimens; participants breathed freely through the mouthpiece up to 20 min in order to collect up to 1.0 mL of EBC.
Malik et al. 2021 [31]	Germany (Hospital)	Prospective Proof of Concept Single center	Convenience sample of 100 EBC samples from 15 hospitalized patients with previous diagnosis of COVID-19	Sens-Abues (Commercial)	Participants breathed through a filter-based device consisting of a mouthpiece, a polymeric electret filter enclosed in a plastic collection chamber. Patients inhale through the nose and tidally exhale 20 times though the mouthpiece.

**Table 3:** A summary of the diagnostic performances of the studies included in the present meta-analysis (Notes: TP = true positive; FP = false positive; FN = false negative; TN = true negative; Se. = sensitivity; Sp. = specificity; PPV = predicted positive value; PNV = predicted negative value; Cohen’s Kappa values should be interpreted as follows: 0.0–0.20 no agreement, 0.21–0.39 minimal agreement, 0.40–0.59 weak agreement, 0.60–0.79 moderate agreement, 0.80–0.90 strong agreement, >0.90 almost perfect agreement).

Reference	No. of Samples	TP	FP	FN	TN	Se.	Sp.	PPV	PNV	Accuracy	Cohen’s Kappa
Ryan et al. 2021 [4]	25	15	0	1	9	93.8%	100%	100%	90.0%	96.0%	0.915
Maniscalco et al. 2021 [14]	105	12	1	1	91	92.3%	98.9%	92.3%	98.9%	98.1%	0.912
Li et al. 2021 [17]	39	3	0	7	29	30.0%	100%	100%	80.6%	82.1%	0.389
Viklund et al. 2022 [18]	36	7	2	16	11	30.4%	84.6%	77.8%	40.7%	50.0%	0.122
Sawano et al. 2021 [16]	39	16	0	24	0	38.5%	-	-	-	-	-
Loconsole et al. 2022 [13]	17	1	0	16	0	5.9%	-	-	-	-	-
Malik et al. 2021 [31]	100	70	0	30	0	70.0%	-	-	-	-	-

**Table 4:** A summary of the diagnostic performances of the RT-qPCR performed on EBC compared to conventional tests on nasal swabs that were included in the systematic review (note: DOR = diagnostic odds ratio; 95%CI = 95% confidence intervals).

	Value	95%CI
Sensitivity	69.5%	26.8 to 93.4
Specificity	98.3%	87.8 to 99.8
Positive Likelihood Ratio	23.608	20.752 to 26.464
Negative Likelihood Ratio	0.433	0.384 to 0.482
Positive Predictive Value	92.5%	91.2% to 93.8%
Negative Predictive Value	77.6%	74.3% to 80.8%
Accuracy	81.5%	78.7% to 84.3%
DOR	51.8	3.276 to 821.554
Cohen’s K Score	0.585	0.535 to 0.635

## Data Availability

Data are available on request to the Corresponding Author.
